# Classification of gluteal muscle contracture in children and outcome of different treatments

**DOI:** 10.1186/1471-2474-10-34

**Published:** 2009-04-07

**Authors:** Chen-Guang Zhao, Xi-Jing He, Bin Lu, Hao-Peng Li, Dong Wang, Zhen-Zhong Zhu

**Affiliations:** 1 Department of Orthopaedic Surgery, The 2nd Affiliated Hospital of Medical College, Xi'an JiaoTong University, Xi'an, Shaanxi, 710004, PR China

## Abstract

**Background:**

Gluteal muscle contracture (GMC) is a clinical syndrome due to multiple etiologies in which hip movements may be severely limited. The aim of this study was to propose a detailed classification of GMC and evaluate the statistical association between outcomes of different management and patient conditions.

**Methods:**

One hundred fifty-eight patients, who were treated between January 1995 and December 2004, were reviewed at a mean duration of follow-up of 4.8 years. Statistical analyses were performed using X^2 ^and Fisher's exact tests.

**Results:**

Non-operative management (NOM), as a primary treatment, was effective in 19 of 49 patients (38.8%), while operative management was effective in all 129 patients, with an excellence rating of 83.7% (108/129). The outcome of NOM in level I patients was significantly higher than in level II and III patients (*P *< 0.05). The results of NOM and operative management in the child group were better than the adolescent group (*P *< 0.05). Complications in level III were more than in level II.

**Conclusion:**

NOM was more effective in level I patients than in level II and III patients. Operative management was effective in patients at all levels, with no statistical differences between levels or types. We recommend NOM as primary treatment for level I patients and operative management for level II and III patients. Either NOM or operative management should be carried out as early as possible.

## Background

Gluteal muscle contracture(GMC), reported by Valderrama for the first time, is a clinical syndrome pathologically-characterized by degeneration, necrosis, and fibrosis of the gluteal muscles and fascia, leading to serious limitation of hip movements [1]. Either congenital or acquired, GMC is not uncommon and exists worldwide, involving the US, France, Italy, Poland, Australia, Spain, China, and India [2-9].

Although numerous reports have outlined the clinical features and surgical treatments for GMC, thus far the classification of GMC, as well as the association between outcome and patient conditions, has not been described.

The purpose of this study was to propose a detailed classification of GMC and evaluate the statistical association between outcome of different management and patient conditions, including severity, pathologic type, and age by reviewing 158 of our patients over the past 10 years.

## Methods

### Study population

Between January 1995 and December 2004, 172 patients diagnosed with GMC were treated in our department. One hundred fifty-eight patients (males, 83; females, 75) 4–17 years of age, were followed for 3–8 years (average, 4.8 years). All of our studies were permitted by the Ethical Board of the 2nd Affiliated Hospital of the Medical College of Xi'an JiaoTong University, and written consent for publication was obtained from the patients or their relatives.

### Classification

**Figure 1:**
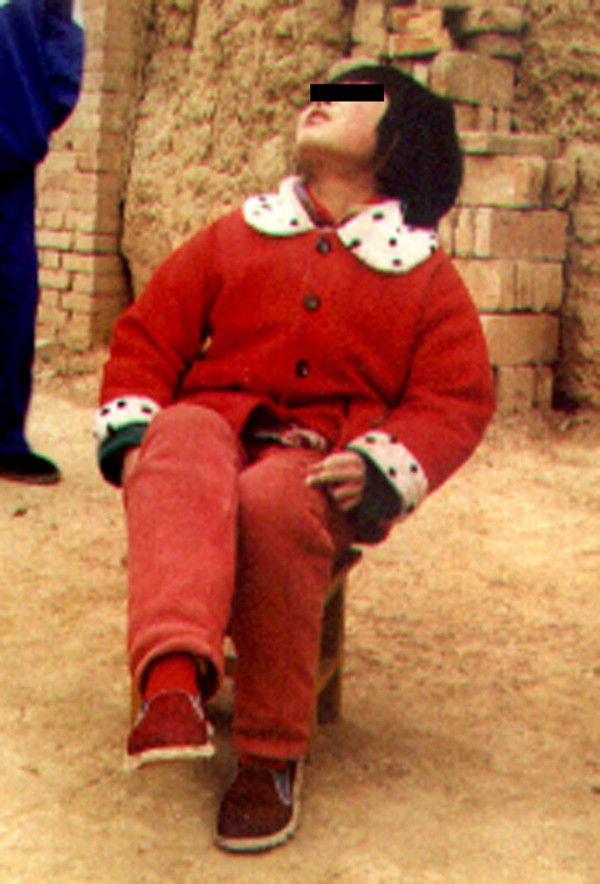
**This figure shows the functional view of a level I patient; she cannot cross her legs only**.

**Figure 2:**
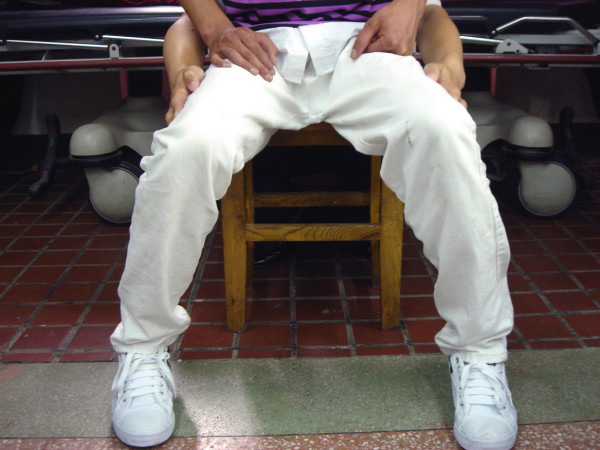
**View of Level II patient**. The abduction contracture angle is 45° with both hip and knee joint in 90°. This patient came to our hospital for the second surgery due to the incomplete release of the contraction band in the other hospital. The huge scaring in the incision can also been seen.

**Figure 3:**
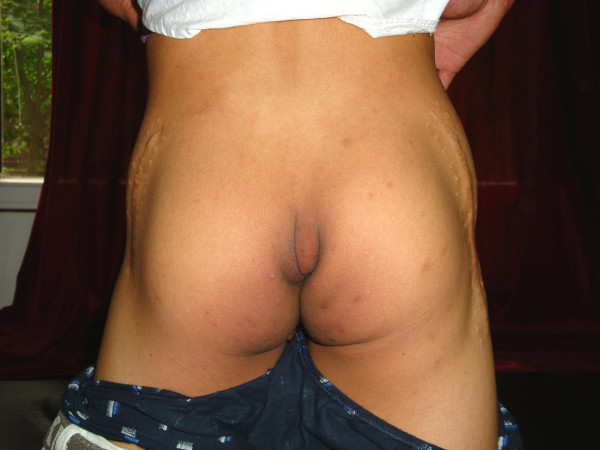
**Level II patient form figure 2, The abduction contracture angle is 45° with both hip and knee joint in 90°**. This patient came to our hospital for the second surgery due to the incomplete release of the contraction band in the other hospital. The huge scaring in the incision can also been seen.

**Figure 4:**
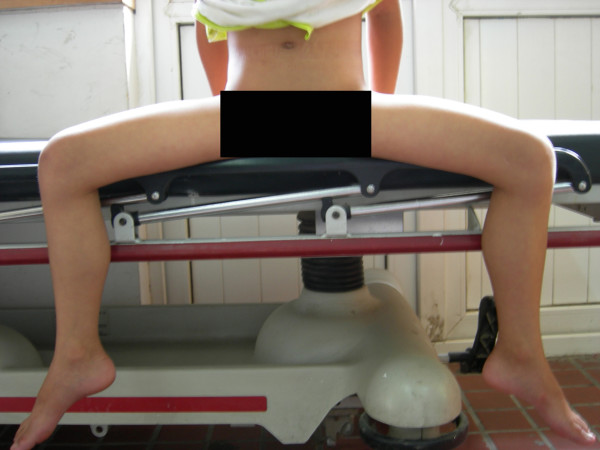
**Level III patient, The abduction contracture angle is more than 60° with both hip and knee joint in 90°**. This patient also has abduction disorders when the hip joint is not in flexion.

**Figure 5:**
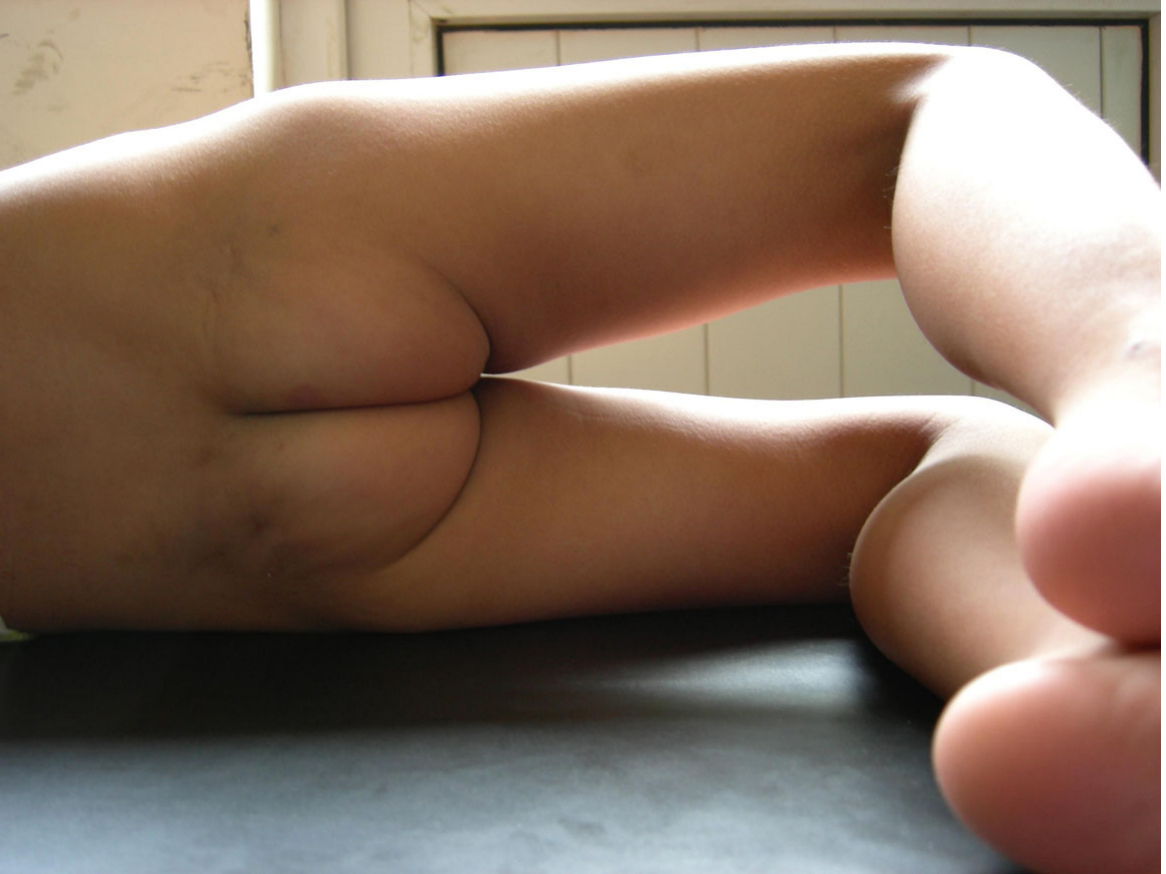
**Level III patient from figure 4, The abduction contracture angle is more than 60° with both hip and knee joint in 90°**. This patient also has abduction disorders when the hip joint is not in flexion.

Based on clinical manifestations and anatomic changes, we propose a classification scheme consisting of 3 levels and 3 types (Table 1; Figures 1, 2, 3, 4, 5).

**Table 1:** Classification of gluteal muscle contracture

Category according to level	
Level I (mild)	The extorsion of lower limb is mild, the abduction contracture is less than 15° with both hip and knee joint in 90° of flexion or adduction range is less than 20° with no flexion. Ober's sign and frog squatting sign is weakly positive. The limp gait is not apparent with lateral inclination of pelvis on anteroposterior radiograph being less than 10°
Level II (moderate)	The extorsion of lower limb is moderate, the abduction contracture ranges from 15° to 60° with both hip and knee joint in 90° of flexion or adduction range is less than 10° with no flexion. Ober's sign and frog squatting sign is positive. The limp gait is apparent with lateral inclination of pelvis on anteroposterior radiograph being less than 20°
Level III (server)	The extorsion of lower limb is severe, the abduction contracture is more than 60° with both hip and knee joint in 90° of flexion or adduction range is less than 0° with no flexion. Ober's sign and frog squatting sign is strongly positive. The limp gait is remarkably apparent with lateral inclination of pelvis on anteroposterior radiograph being more than 20°

Category according to type	

Type MA	Gluteus maximus contraction type
Type MEI	Gluteus medius and minimus contraction type
Type AGM	Gluteus maximus, medius and minimus contraction type (All gluteal muscle contraction type)

### Treatments

#### Non-operative management (NOM)

Massage and physiotherapy, such as shortwave diathermy and hot packs, were the basic NOM techniques for patients. Following NOM, the patients could engage in the following exercises: squats with the knees close to each other, walking on a line, and moving the hips with effort. Active and passive exercise methods should be undertaken and NOM should be performed for at least last one-half year.

#### Operative management

**Figure 6:**
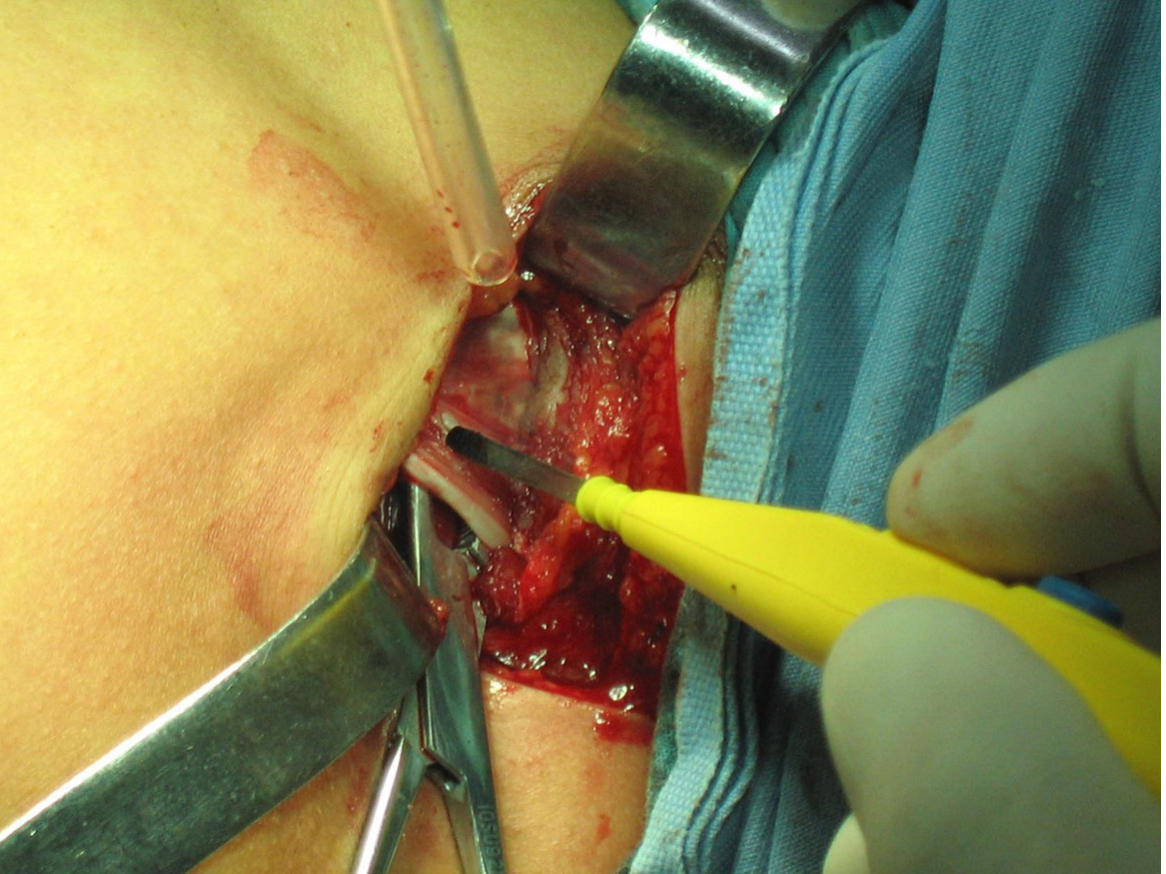
**Intraoperative view of the lesion of contraction**.

With the patient in a lateral position, a skin incision (averaging 8 cm) was made at the lower part of a line between the trochanter major and the posterosuperior iliac spine. The soft tissue was dissociated until the contraction region of the gluteus maximus, which mainly existed in its upper one-half, was observed. After the gluteus maximus and the associated fascia were released, the patient was examined to determine whether the Ober's sign was positive, and if so, the gluteus medius and gluteus minimus were explored and released as well (Figure 6). Sometimes, severe cases involve even deeper parts, such as musculus piriformis, obturator internus, and the articular capsule, in which case, it should also be released and reconstructed. The sciatic nerve should be carefully protected when the deeper muscles are being released. A drainage piece or tube was placed routinely. Exercises should be carried out 24~48 hours after the operation.

### Evaluation after treatment

Patients who completely recovered were considered to have excellent results for level I or an adduction angle that improved > 45° for level II, and 60° for level III. Failure indicated that the situation was not improved or even worse, and patients needed another operative intervention. Fair result would be improved, but were not excellent.

### Statistics

Statistical analyses were performed using X^2 ^test and Fisher's exact test. A *P *value < 0.05 was considered statistically significant.

## Results

There were 158 patients (males, 83; females, 75) diagnosed with GMC included in this study. The types of GMC were MA (n = 108, 68.4%), MEI (n = 15, 9.5%), and AGM (n = 35, 22.1%). The levels of GMC were I (n = 34, 21.5%), II (n = 85, 53.8%), and III (n = 39, 24.7%); (Table 2).

**Table 2:** Gluteal muscle contracture cases according to level and type

	MA	MEI	AGM	Total
Level I	28	1	5	34 (21.6)
Level II	61	8	16	85 (53.8)
Level III	19	6	14	39 (24.6)

Total	108 (68.4)	15 (9.5)	35 (22.1)	158 (100)

Values are number (percentage).

All 34 level I patients, 12 of 85 level II patients, and 3 of 39 level III patients received NOM as primary treatment, which was effective in 19 patients (38.8%; Table 3). The outcome of NOM in level I patients was significantly higher than in level II and III patients (P < .05). The effective rate of NOM in the level I group of children (13/19 [68.4%]) was higher than in the adolescent group (5/15 [33.3%]; *P *< .05). Operative treatment was performed in 6 level I patients, 84 of 85 level II patients, and all level III patients. All patients had excellence or fair results, while none failed. The excellence rate was 83.7%, with no statistical difference (Table 4). The excellence rate of operative management in the child group (63/129 [48.8%]) was higher than in the adolescent group (66/129 [51.2%]; *P *< .05). No difference was seen between the different types with NOM or operation.

**Table 3:** Outcome of nonoperative management

	Effective	Failed	Effective rate
Outcome according to level			
Level I	18	16	18/34 (52.9)
Level II	1	11	1/12 (8.3)
Level III	0	3	0/3 (0.0)
Total	19	30	19/49 (38.8)

Outcome according to type			
MA	17	22	17/39 (43.4)
MEI	0	2	0/2 (0.0)
AGM	2	6	2/8 (25.0)
Total	19	30	19/49 (38.8)

Values are number (percentage).

**Table 4:** Outcome of operative management

	Excellent	Fair	Excellent rate
Outcome according to level			
Level I	6	0	6/6 (100)
Level II	71	13	71/84 (84.5)
Level III	31	8	31/39 (79.5)
Total	108	21	108/129 (83.7)

Outcome according to type			
MA	70	13	70/83 (84.3)
MEI	13	2	13/15 (86.7)
AGM	25	6	25/31 (80.6)
Total	108	21	108/129 (83.7)

Values are number (percentage).

Complications after operative management only appeared in level II and III patients, and included scar swelling (II = 16; III = 48 [some severe cases exceeded 0.7 cm]), hematomas (III = 4), infections (II = 1; III = 1), and wound dehiscence (III = 1).

## Discussion

Although several factors might be the cause of GMC, we found that repeated injections in the buttocks was perhaps the main cause from our previous epidemiologic survey, which was consistent with other reports [9,10]. For all of the patients in this study, 108 parents of the patients recalled repeated injection in the buttocks of their children at an early age; 5 parents denied injections in the buttocks, and were thus idiopathic just as some other reports have suggested and it would take more effort for confirmation [11,12]. Two patients appeared to have a genetic tendency, as both the children and their fathers were diagnosed with GMC, although this possibility also needs further work to be identified. One patient was diagnosed with a fibroma in the left hip joint. The diagnosis of GMC mainly depends on anamnesis, and typical clinical manifestations; X-ray and MRI studies would not be requisite, but could always help eliminate other osteopathologic changes and make available additional information before surgery [13,14].

Reasonable classification would help us to achieve a better understanding of pathologic features of this disease and indicate more suitable treatments. Previous studies have mainly focused on contracture of the gluteus maximus, which always included the most severe cases, or created a classification emphasizing the aesthetic aspects more than the functional aspects, and which would be helpful to plastic surgeons [11,15,16]. However, our classification system was based on all types of contractures with different levels focusing on functional and pathologic changes. Out of concern regarding clinical manifestations and anatomic changes, we proposed a classification scheme consisting of 3 levels and 3 types. Type MA only involves the gluteus maximus, so patients with this type would represent extorsion of the lower limb, adduction disorders when the hip joint is flexed, especially while sitting and squatting and with a limitation of flexion. The gluteus medius and minimus play an important role in abduction of the hip and it had functions of both extorsion and intorsion. Based on the location in which muscle injections are usually performed, it would only result in an extorsion abnormity. Therefore, contracture of the gluteus medius and minimus (type MI) would be in extorsion abnormalities, and adduction disorders, even without flexion and limp gait when the lesions on the two sides are not in parallel. Some patients with limp gait would appear to have an incline of the pelvis and compensatory scoliolosis. However, some reports have revealed that the lesion of their patients were only limited to the gluteus medius or gluteus minimus, leading to a disorder of limp gait, but we did not encounter such [17]. For type AGM, both two types of the clinical manifestations would appear.

Since Valderrama reported GMC in 1970, surgeons have deemed that operative management is an effective way to cure the disorders, but in terms of NOM, most reports have concluded it to be invalid [18]. However, considering the patient's condition, which they cured and was relatively severe from the description, we still hold the opinion of giving a chance for patients with trivial function disorders. Thus, for all level I patients, we adopted a series of physiotherapies of NOM preferentially, and operative management would be offered afterwards in case that the situation did not improve or they insisted on better results [Table 5]. NOM was effective in one-half of the level I patients (52.9%), but the situation in all effective cases was just improved, not totally recovered. This suggested the limitation of NOM in curing patients. However, with respect to the financial costs to some poor families, the mental stress of the child, and previous conditions, most parents and children themselves were satisfied with the outcomes. Those patients in whom the condition was not improved had been offered operative management afterwards (10 patients refused), and they all totally recovered. For patients in levels II and III, we give patients operative management directly. However, because of education and other matters, 12 patients in level II and 3 patients in level III postponed their operation. During the interval, they had NOM instead (approximately one-half year), and only 1 slightly improved, while the others totally failed. This result was significantly lower than the result of NOM in level I patients (*P *< 0.05). Nevertheless, the function of hip movement in level II and III patients with operative management improved considerably. Seventy-one of 84 level II patients and 31 of 39 level III patients got excellent results. This result, together with the result in NOM, indicated the necessity of operative management in levels II and III. The outcome of operative management had no statistical difference between the 3 levels and between the 3 types, which indicated us that operative management would be a perfect choice for any level or type of GMC patients. We found age was one important factor that influenced the results of both NOM and operative management. Patients in the child group gained better results than the adolescent group in both the treatments, which suggested the importance of earlier medical intervention.

**Table 5:** Recommendation of treatment strategy

Levels of Patients	Recommendation of treatment strategy
Level I	NOM as first choice, operation as recruitment treatment. Interogluteal incision or use arthroscopic technique is recommended.
Level II	Operative management as first choice, NOM as choice for recruitment treatment for waiting for operation or after operation. Surgeons could adopt interogluteal incision or use arthroscopic technique depending on the condition of patients.
Level III	Operative management as first choice, NOM as choice for recruitment treatment for waiting for operation or after operation. Operation under direct-viewing with conventional incision is commended, in order for complete releasing.

The success of operative management rested in two points: 1) complete release of fibrotic tissue and 2) protection of intact tissue. Incomplete releasing would result in incomplete disappearance of complaints and physical sign, while excessive releasing would harm the stability of the hip joint which might lead to Trendelenburg gait. Six patients came to our department for a second operation and 2 patients for a third procedure; all of them were due to incomplete release. It was very important to check whether the Ober's sign is positive intraoperatively, especially in type MEI and AGM.

**Figure 7:**
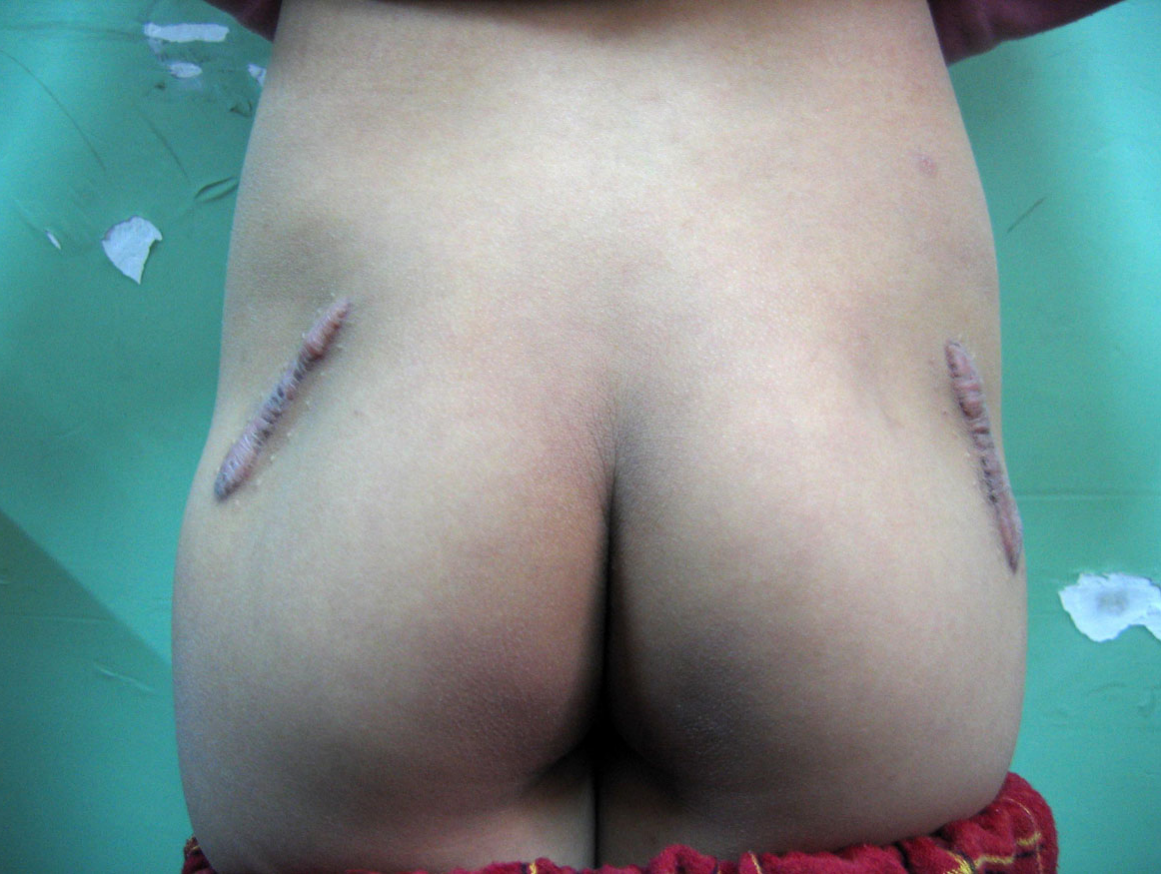
**Hypertrophic scar of the incision in a GMC patient**.

Until now, the operative strategy included a Z-plasty and simple releasing. Of all patients included in this study, only simple releasing operations were performed, thus further research is needed regarding which operative method is better. The most frequent complication was swelling of hypertrophic scars or keloids; 64 patients appeared visible (7 mm at most), which was in agreement with some other reports (Figure 7) [15,19]. Probable solutions to this problem would possibly be an intergluteal incision or using an arthroscopic technique [16,20]. As fibrosis of the gluteal muscle in GMC patients share the same pathologic changes with hypertrophic scars or keloids, that is, hyperplasia of fibrocytes and increased synthesis of extracellular matrix (ECM), including collagens. Thus, the association between fibrosis of the gluteal muscle and formation of hypertrophic scars or keloids in the incision need more research.

## Conclusion

Our work is a retrospective study of gluteal muscle contractures in children. We proposed a detailed classification of GMC and have assessed the outcomes of different management techniques. NOM was more effective in level I patients than in level II and III patients. Operative management was valid in patients at all levels with no statistical difference. We recommend NOM as primary treatment for level I patients and operative management for level II and III patients. Age was one important factor which influenced the final result, which suggested that either NOM or surgery should be carried out as early as possible.

## Competing interests

The authors declare that they have no competing interests.

## Authors' contributions

All authors read, edited, and approved the final manuscript. CGZ and XJH are the lead investigators, and developed the design of the study, carried out data-acquisition, analysis, interpretations, and prepared as primary authors for the manuscript. BL, HPL, and DW were responsible for the design, project supervision, and writing of the manuscript. ZZZ assisted in carrying out data acquisition and was involved in preparing the study design and in writing the manuscript.

## Pre-publication history

The pre-publication history for this paper can be accessed here:



## References

[B1] Valderrama JAF (1970). A cause of limited flexion and adduction of the hip in children. J Bone Joint Surg [Br].

[B2] Howard RC (1971). Iatrogenic quadriceps and gluteal fibrosis. J Bone Joint Surg [Br].

[B3] Chigot PL, Vialas M (1969). Fibroses rétractiles du moyen fessier. Rev Chir Orthop.

[B4] Motta A (1971). La miosite fibrosa causa di limitazione articolare del ginocchion e dell'anca. Clin Ortop.

[B5] Henvkovský O (1961). Progressive fibrosis of the vastus intermedius muscle in children. A cause of limited knee flexion and elevation of the patella. J Bone Joint Surg [Br].

[B6] Williams PF (1968). Quadriceps contracture. J Bone Joint Surg [Br].

[B7] Fenollosa Gomez J, Quiles Galindo M (1969). Retracción bilateral del gluteo mayor. Reo Ortop Traumat.

[B8] Karlen A (1964). Congenital fibrosis of the vastus intermedius muscle. J Bone Joint Surg [Br].

[B9] Mehta MH (1972). Bilateral congenital contracture of the ilio-tibial tract. J Bone Joint Surg [Br].

[B10] Hang YS (1979). Contracture of the hip secondary to fibrosis of the gluteus maximus muscle. J Bone Joint Surg Am.

[B11] Brignall CG, Brown RM, Stainsby GD (1993). Fibrosis of the gluteus maximus as cause of snapping hip. J Bone Joint Surg Am.

[B12] Shen YS (1975). Abduction contracture of the hip in children. J Bone Joint Surg [Br].

[B13] Cai JH, Gan LF, Zheng HL (2005). Iliac hyperdense line: a new radiographic sign of gluteal muscle contracture. Pediatr Radiol.

[B14] Chen CK, Yeh L, Chang WN (2006). MRI diagnosis of contracture of the gluteus maximus muscle. AJR Am J Roentgenol.

[B15] Shen YS (1982). Gluteus maximus contracture. Clin Orthop Relat Res.

[B16] Gonzalez R (2006). Gluteal Retractions: Classification and treatment techniques. Aesthetic Surg J.

[B17] Yue L, Xinhua P, Kai T (2005). Operative management for children with pelvic obliquity caused by gluteal muscle contracture. Chin J Pediatr Surg.

[B18] Valderrama JAF, Miguel RE (1981). Fibrosis of the gluteus maximus: a cause of limited flexion and adduction of the hip in children. Clin Orthop Relat Res.

[B19] Peiro A, Fernandez CI, Gomar F (1975). Gluteal fibrosis. J Bone and Joint Surg [Am].

[B20] Liu YJ, Wang Y, Xue J (2009). Arthroscopic gluteal muscle contracture release with radiofrequency energy. Clin Orthop Relat Res.

